# COVID-19: not the time for health economists? A plea for more proactive health economic involvement

**DOI:** 10.1007/s10198-021-01349-2

**Published:** 2021-07-13

**Authors:** J.-Matthias Graf von der Schulenburg

**Affiliations:** Leibniz University Hannover, Hannover, Germany

## Health economist on the sidelines

The COVID-19 pandemic does not seem to be the hour for economists. This is very astonishing as the COVID-19 pandemic has an enormous impact on all parts of the economy. Most sectors were hit hard by the direct consequences of the pandemic as well as by the measures to reduce infection rates. Governments jumped in with subsidies at record levels to stabilize economies by increasing national debt in very short time frames and at very significant levels. Looking at the current vaccination campaign, it is all the more astonishing that economists can only be found on the verge of decision-making. Why is that? Two answers are suggested in this editorial. First, economists have so far not yet developed standardized methods for evaluating vaccination. Second, the essence of any economic consideration is an estimate of costs. But precisely these cost estimates are extremely complex and difficult, as there is no transparency about the real costs and spending for the vaccination programs.

Since the beginning of the pandemic, many experts have been heard by decision makers and have spoken out publicly: virologists, epidemiologists, intensive care physicians to name a few. Economists were rather quiet. Detailed analysis of the general consequences of the pandemic on the economy and on public finances were hardly ever presented, nor were the costs and benefits of specific measures systematically examined or publicly debated. This applied both to the many interventions in the health systems as well as to the vaccination campaigns that are now running since the beginning of the year. Obvious questions are: what was the basis of decision making: how were the costs and the benefits of the current initiatives analyzed? And who has evaluated whether this vaccination campaign is organized in the most efficient and/or most cost-effective way?

There is little debate on the economic consequences and the resulting cost benefits from a health economic point of view. Instead, the public interest concentrates mainly on the epidemiological aspects of the pandemic. How do infection incidence and reduplication rates develop? What is the death toll? Are the different pandemic containment measures taken by governments—from the obligation to wear masks to the complete or partial shutdowns of public life—appropriate to achieve the public health goals? What is missing is the debate on total direct and indirect cost, and the efficiency and cost-effectiveness of the implemented measures. Only few questions were raised and studied by economists such as the association between income inequality and COVID-19 reported cases and deaths per-million [1].

## Cost effectiveness of the vaccination programs

Experts from the UK Center for the Mathematical Modeling of Infectious Diseases COVID-19 working group have recently conducted a first estimate on the financial benefit of vaccination programs [2]: “From a health-care perspective, introducing vaccination leads to incremental net monetary values ranging from £12 billion to £334.7 billion in the best-case scenario and from £1.1 billion to £56.9 billion in the worst-case scenario”. Such rough estimates are more likely to create confusion than to increase trust in the contributions of health economists.

Regardless of the cost of the vaccination program, there are management questions that call for economic analysis: who should make which purchasing decisions and how should logistics, storage and administration be organized in the most cost-effective way? In Germany, for example, the question arises as to whether it is more efficient to let all resident doctors and/or vaccination centers carry out the vaccination program.

Economists have developed solid methods to evaluate the cost and benefits of pharmaceuticals. Based on these methods, health technology assessment organizations and pricing and reimbursement agencies have established guidelines for the economic evaluations of drugs. However, the debate of which tools should be used to evaluate and to measure the cost-effectiveness of pandemic vaccination programs is still ongoing. One issue is that many vaccinations do not only protect the vaccinated person, but also provide a social good for the broader society, the most important effect being herd immunity which protects the entire population. In order to analyze these effects over time, complex population models need to be established that map the vaccination process. Only a few members of the pharmaco-economic community have so far dealt with the development of such evaluation methods for vaccination programs (for instance [3]).

In 2018 the ISPOR task force for the economic evaluation of vaccines published its report. Among others, it recommends that the economists evaluating new vaccines should “take into account the decision maker’s policy objectives and country-specific decision context when estimating the uptake rate in the target population over time using a dynamic transmission epidemiological model, assessing vaccination program implementation and operating costs; and the changes in cost and health outcomes of the target disease(s).” [4]. This quote exemplifies the complexity of evaluating vaccination programs.

## Operating cost of vaccination programs

As it is difficult to develop methods and models to fully evaluate vaccination programs from an economic point of view, the ISPOR report recommends starting with an estimation of the operating costs. In a first study project, we have started to assess these costs.

However, even quantifying operating costs is not an easy task as in Europe, governments currently fund the procurement of vaccines and mass immunization programs. Also, vaccination initiatives differ significantly by country in Europe. Surprisingly, operating costs are not transparent despite the fact that they are publicly funded. In Germany for example, the government has so far not published the costs associated with the vaccination program.

Germany, the most populated EU member state, started the vaccination program with the establishment of dedicated vaccination centers, differing by size, organization and region. Since April, GPs are also involved in the vaccination effort and will probably take it over by the end of summer as federal states plan to close vaccination centers in autumn. These decisions were made without knowledge and analysis of operating costs and the efficacy of different options to vaccinate the population. Up till now we have only limited knowledge about the operational cost including the costs of procuring the vaccine, the transport, storage, distribution, processing and administration of the vaccines.

## Operating cost of vaccination centers and GPs

In order to get a first impression of the costs, we carried out a structured survey in the first quarter of 2021. At the time, two mRNA vaccines were continuously administered in these vaccination centers, namely mRNA-1273 (Moderna) and BNT162b2 (Pfizer-BioNTech).

Data were collected through telephone interviews using a structured questionnaire that elicited time spent along the logistics process within the vaccination centers (receipt, storage, thawing, disposal) as well as in the handling, training, preparation, and administration of the vaccine for the two alternatives considered. The length of the interviews was approximately 50 min. 12 interviews were available for analysis: 10 interviews were conducted in Germany (with a regional distribution over seven states), and two in Switzerland.

An unexpected result of the investigation was the realization that the sub-processes of logistics, preparation and administration were characterized by division of labor in which the various steps of the process were executed by different dedicated personnel. Interview responses about preparation and administration of the vaccines were generally more frequent and more accurate than comments on the sub-processes in logistics.

Parallel to the interviews, a cost model was designed that evaluated the observed resource utilization in terms of material and staff time, and applied a weighting that allowed a cost comparison of the sub-processes. We incorporated these results into an overall view that included the daily costs of the vaccination centers for the processes considered (logistics, preparation, administration). In addition to testing the model with respect to individual time and cost parameters in the context of sensitivity analyses, the model offered the possibility of simulating economies of scale (both positive and negative) by assessing vaccination centers of different volumes: 200, 600, 1500 doses per day, corresponding to the population density in the respective region. To also estimate the impact of the vaccination program rollout in the setting of GP practices in Germany, we modeled the resource utilization taking the assumption of 20 daily doses per practice. This assumption corresponds with first observations from the GP rollout and gains relevance in light of future booster vaccination programs which will be implemented in GP practices.

We modeled the direct cost data assuming hourly total labor cost of € 40 for the medical/pharmaceutical assistants. The results are shown in the Table 1.

**Table 1:** Processing cost of vaccination by type of vaccine and size of vaccination unit

Center size	Resource utilization/dose	
	mRNA-1273	BNT162b2
Centers with 200 vaccinations/day	€ 0.56	€ 1.09
Centers with 600 vaccinations/day	€ 0.54	€ 1.03
Centers with 1500 vaccinations/day	€ 0.53	€ 1.00
GP office	€ 0.88	€ 1.74

In summary, we found that first, direct vaccination costs vary between the two different mRNA vaccines. This difference is mainly driven by the need to constitute the BNT162b2 vaccine. Second, the operating costs decrease with the size of the vaccination unit due to economies of scale in larger vaccination operations.

Other results include the observation that the proportion of resource utilization required for logistic sub-processes (handling receipt, storage, thawing) is about 2% in large centers. This proportion increases to 4% in medium-sized centers and to 8% in small centers. In GP practices, the logistic sub-processes requires 41% of the total resource utilization. It is not surprising that the results are most sensitive to a variation of the labor cost and vaccine constitution time.

As our study only estimates the processing costs directly related to the vaccination and not the total running cost of a vaccination center or GP office and the cost of the vaccine itself, it can only be a first and small step in determining the costs of COVID-19 vaccination programs. These costs only represent a small part of the total costs. Overhead costs for the ongoing operations in vaccination centers were not included. Applying the results to vaccinations in GP offices highlighted the potential for “negative” economies of scale, particularly in the area of logistics. This is consistent with the results of studies on vaccinations in the pediatric context. These studies showed that logistics and organizational costs prior to actual vaccination in physicians’ practices account for approximately 50% of the total time spent [3, 5]. However, it remains to be examined in detail to what extent the assumptions regarding the process also apply to GP practices and to refine the model considering this perspective.

One may ask: do these differences in the operating cost of different vaccination options matter? The answer is no and yes. No, because we only focused on one special, and comparatively easy to quantify, aspect of the overall cost associated with the COVID-19 vaccination. The total costs are much higher and the single cost components are covered by different payers. Vaccines are currently bought and financed by the government. The vaccination units receive a per capita fee or a fixed budget from the government. GPs receive a fee-for-service from the sick funds or health insurers. Our model shows that the vaccine preparation costs appear relatively small for one single dose and that the associated administrative cost of the vaccination units will most likely be higher.

But to come back to our initial question: do differences in operating cost for vaccinations matter? Yes, because previous cost studies of immunization show that even individual elements of the overall process can have a significant budgetary impact in the context of nationwide immunization programs. Using a calculation model, [6] evaluated different approaches to skin preparation in the context of COVID-19 vaccinations and arrived at a budget effect of up to USD 600 million for the USA if the frequently debated pretreatment of the skin by alcohol is omitted. Even small cost differences matter as they add up to substantial amounts of money, especially as tens or hundreds of millions of people need to be vaccinated to achieve herd immunity.

## Lessons learned

All available vaccination options for COVID-19 are very effective. Some may even be more effective than others and may also have different health economic profiles. The methodology to measure the benefits of vaccination programs especially when they have herd immunity as a goal are currently being debated. In this editorial, I have only touched on one direct cost aspect. To become a proactive discussion partner in the design of vaccination programs, the health economic community should invest more time to develop consistent methods for the economic evaluation of vaccines and vaccination programs, including indirect effects. As a first step, health economists should agree on a methodology to determine the perspective of economic analysis and the elements of quantifying the benefits and their associated costs. Then, pandemic times will also be the time for health economists.
